# Study on viscosity and yield stress of magnetic fluid applied to low temperature magnetic fluid seal

**DOI:** 10.1038/s41598-025-26343-z

**Published:** 2025-12-02

**Authors:** Jiawei Liu, Decai Li

**Affiliations:** 1 China Nuclear Power Engineering Co., Ltd, Beijing, 100032 China; 2 State Key Laboratory of Tribology in Advanced Equipment, Beijing, 100084 China

**Keywords:** Magnetic fluid, Magnetic fluid seal, Rheology, Viscosity, Yield stress, Starting torque, Engineering, Materials science, Nanoscience and technology

## Abstract

Magnetic fluid seal is one of the most mature applications of magnetic fluid and is widely used in numerous fields. However, magnetic fluid seals used in low-temperature environments often encounter the problem of high starting torque. The rheometers commonly employed to characterize the rheological properties of magnetic fluid seal are limited by their temperature range, making it challenging to evaluate the properties of magnetic fluid seal under extremely low temperatures (such as -55 °C). Meanwhile, the standing time on the yield stress of magnetic fluid or the starting torque of magnetic fluid seal in low-temperature should be added to characterization especially for analyzing the magnetic fluid seal in low temperature. Additionally, the commonly used Herschel-Bulkley (H-B) model struggles to accurately fit the static yield stress of magnetic fluid seal. To address this issue, a stress-strain method was proposed to fit the static yield stress of magnetic fluid seal, and empirical formulas for viscosity and yield stress were developed. The effects of temperature, magnetic field, and resting time on yield stress were analyzed, and the starting torque of magnetic fluid seals under specific operating conditions was calculated. The results obtained from this method will provide valuable guidance for engineering applications.

## Introduction

Magnetic fluids are suspensions consisting of nanosized ferromagnetic particles dispersed in a carrier liquid. The ferromagnetic particles used in magnetic fluid seal are single-domain and typically range from 5 to 15 nanometers in size^1^. These particles are coated with surfactants or polymer molecules, which act like springs to repel nearby particles and prevent them from aggregating. This ensures a stable dispersion of the particles within the carrier liquid. In 1965, Stephen from NASA^2^ successfully synthesized the first stable magnetic fluid, sparking subsequent research into various properties of magnetic fluid seal, including their suspension characteristics^3,4^, rheological properties^5,6^, magnetization behavior^7^, magnetoviscous effect^8,9^, magnetothermal effect^10,11^, and magneto-optical effect^12,13^. magnetic fluid seal have extensive industrial applications, with over 170 fields utilizing them, many of which cannot be substituted by other materials^14^.

However, more and more industrial standards have expanded the demarcation range for low temperatures, specifying that the minimum threshold should be -55 °C. The starting torque of magnetic fluid seal increases as the temperature decreases, which negatively affects the normal operation of the device. M. Kubík et al.^15^ proposed a novel sealing structure that uses an electromagnet as the magnetic source. By altering the magnetic circuit, the arrangement of magnetic particles is modified to reduce friction torque. However, compared to traditional sealing structures, this design significantly reduces both pressure resistance and axial space utilization by more than half. Since this approach lowers torque at the expense of sealing pressure resistance, it has not been widely adopted. Y. Cheng^16^ conducted experimental research on the starting torque of magnetic fluid seal at room temperature under different resting times. The results showed that as the resting time increased, the starting torque gradually increased until reaching a stable state. This research reflects the important influence of standing time on the yield stress of magnetic fluid or the starting torque of magnetic fluid seal. It’s a pity that most researches devoting on the rheological characters of magnetic fluid don’t consider on the standing time. J. Wu^17^ studied the issues of high friction torque in magnetic fluid seal under low-temperature conditions and analyzed the changes in pressure resistance and torque through experiments. The results indicated that the pressure resistance of magnetic fluid seal increases with the amount of magnetic fluid injected until reaching the structure’s maximum pressure capacity, after which further injection does not enhance sealing capacity. However, the starting torque continues to increase with the volume of injected magnetic fluid. His research concluded that injecting an optimal amount of magnetic fluid can reduce magnetic fluid seal starting torque. Despite these researches, existing methods either provide only qualitative strategies to reduce starting torque or suffer from significant flaws, failing to propose an effective way to calculate the starting torque in low-temperature rather than addressing the problem of high starting torque. L. Li^18^ synthesized a silicone oil based magnetic fluid that retains fluidity at -40 °C. Rheological characterization demonstrated that the prepared magnetic fluid’s flowability, viscosity, and yield stress were minimally affected by temperature changes. Experimental results further confirmed that this magnetic fluid could reduce the starting torque of magnetic fluid seals in low-temperature environments. However, many magnetic fluid seal lose fluidity under such conditions, leading to seal failure.

In the study of magnetic fluid seals for high-precision equipment, it is crucial to maintain low and stable torque and pressure resistance during cycles of startup and shutdown, as well as under low-temperature conditions. Some magnetic fluid seal that are not resistant to low temperatures or have high viscosity tend to exhibit significantly increased yield stress and viscosity under such conditions. This results in abrupt changes in rotational and starting torque, severely impacting the precision of the equipment. Therefore, it is essential to characterize the rheological properties of magnetic fluid seal to predict and compare their behavior under targeted operational conditions.

### Main factor

*B* represents the magnetic field, *D* represents the shear rate, *T* represents the temperature and *σ*_s_ represents the yield stress, ${K_\tau }$ is the stress factor, and ${K_\gamma }$ is the strain factor, $\tau$ is the shear stress (Pa), $\gamma$ is the shear strain, $\eta$ is the viscosity of the magnetic fluid (Pa·s), and *t* is time (s), radius r1 can be approximated as the flow radius and $\eta$ is the magnetic fluid viscosity ${\eta _c}$ represents the viscosity of the carrier fluid at room temperature, C is constant number, ${k_0}$ is the Boltzmann constant, *m* represents the maximum magnetic moment of the magnetic particle. In the equation for each magnetic particle, there is $m={M_d}{V_p}$, where ${V_p}$ is the volume of each magnetic particle and ${M_d}$ is the saturation magnetization of the solid magnetic particle.

## The preparations and rheological characterization of magnetic fluid

### The preparations of low temperature resistant magnetic fluid

One of the key components in magnetic fluid seal is the magnetic fluid. If the magnetic fluid can keep fluidity in low temperature, the carrier fluid must have a low freezing point. Here, PAO-3.5with − 78 °C freezing point is chosen as the carrier fluid of low temperature resistant magnetic fluid. PAO is the abbreviation of poly-α-olefin (Poly-Alpha-Olefin). 3.5 represents its kinematic viscosity value at 100℃ (3.5 mm²/s) The preparation method for low temperature resistant magnetic fluid is detailed as follows: A mixture with a mass ratio of Fe^3+^:Fe^2+^ = 1.75:1 is dissolved in 300 ml deionized water. The solution is placed in a water bath and heated with stirring at 45 °C and 400 rpm. Once Fe^3+^ and Fe^2+^ are completely dissolved, excess ammonia is slowly added. The reaction proceeds for 45 min, and the solution is then washed with deionized water until the pH reaches 7. Subsequently, 300 ml deionized water is added and the solution is placed in a water bath, and heated with stirring at 85 °C and 400 rpm. Oleic acid (OA, C_18_H_34_O_2_) is added dropwise, and the reaction continues for 1–1.5 h. Excess water is removed using a magnetic separation method, and the mixture is then dried in a vacuum oven. The dispersant Polyisobutylamine (PIBA) is used, and after drying the magnetic powder, the mass ratio of PIBA: Fe_3_O_4_@OA: PAO-3.5 is set at 1:6:6. The mixture is ground in a vacuum ball mill for 2 h to obtain low temperature resistant magnetic fluid with a mass fraction of 45wt%. The raw materials required for the preparation of Low-temperature resistant magnetic fluid is shown in Table 1.

**Table 1:** Raw materials required for the Preparation of Low-temperature resistant magnetic fluid.

Name	Purity	Purchase source	Relative molecular mass/density
FeCl_2_·4H_2_O	99.95%	Shanghai McLean Biochemical Technology Co., LTD	198.81
FeCl_3_·6H_2_O	99%	Shanghai McLean Biochemical Technology Co., LTD	270.3
NH_3_·H_2_O	25 wt%	Shanghai McLean Biochemical Technology Co., LTD	0.91 kg/m³
C_18_H_34_O_2_, Oleic acid(OA)	AR	Sinopharm Chemical Reagent Co., LTD	282.47
PAO-3.5	99%	Shenzhen Huashengyuan Petroleum Technology Co., LTD	795 kg/m³
PIBA	99.9%	AVIC New Materials Co., LTD	900 kg/m³

**Fig. 1:**
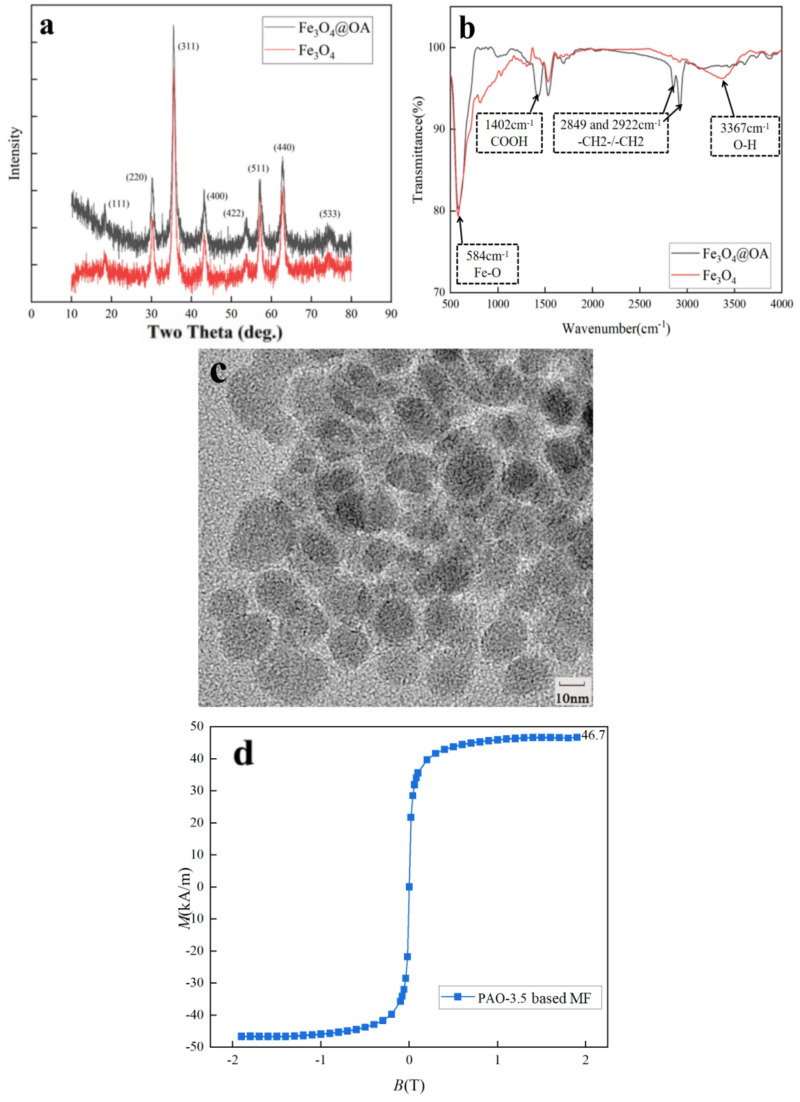
(**a**) XRD pattern of bare Fe_3_O_4_ and Fe_3_O_4_@OA particles (**b**) FT-IR spectra of bare Fe_3_O_4_ and Fe_3_O_4_@OA particles (**c**) TEM image of Fe_3_O_4_@OA particles (**d**) The magnetic fluid magnetization curve of PAO-3.5 magnetic fluid obtained through VSM characterization.

Subsequently, X-ray diffraction (XRD), Fourier transform infrared spectroscopy (FT-IR) and transmission electron microscopy (TEM) were employed to characterize the dispersion and coating of magnetic particles, base carrier liquid, and the final magnetic fluid. XRD was measured by Anton Paar XRDynamic 500, FT-IR was measured by JASCO FT/IR-6000, and TEM was measured by Thermo Fisher Talos L120C. XRD characterization was conducted on bare Fe_3_O_4_ particles and OA-coated Fe_3_O_4_ nanoparticles (Fe_3_O_4_@OA). The results, as shown in Fig. 1(a), revealed that the peak positions and diffraction patterns of both particle samples align with the inverse spinel crystal structure (JCPDS 19–0629). According to the results of similar peak positions and diffraction intensities of peaks of two kinds of particle samples, the coating process is the chemical reaction between outer layers of surfactants and Fe_3_O_4_. The inner crystal structures remain the same after the coating process. The particle size, estimated using the Scherrer equation, is approximately 10 nm, aligning with existing research results^6,8^.

Subsequently, we characterized bare Fe_3_O_4_ particles and Fe_3_O_4_@OA particles using FT-IR, with the results presented in Fig. 1(b). Both samples exhibit absorption peaks at 570 cm ^− 1^, attributed to the Fe-O bonds between Fe and O atoms^23^. At 1402 cm ^− 1^, the absorption peaks arises from the COOH group of OA. The two absorption peaks at 2800–3000 cm ^− 1^, respectively correspond to the asymmetric and symmetric stretching of the methylene groups^24^. The disappearance of the “-OH” functional group at 3400 cm ^− 1^ for Fe_3_O_4_ particles after encapsulation by OA suggests that hydroxyl groups serve as anchoring points in OA that bind with Fe_3_O_4_ particles. These four absorption peaks collectively confirm the presence of an organic coating on the surface of Fe_3_O_4_ particles.

Observing the TEM image in Fig. 1(c), we note that the particles are well-dispersed, and the majority of magnetic particles exhibit a spherical morphology, with an average particle size of approximately 10 nm.

Subsequent to this, the magnetization properties of PAO−3.5 based magnetic fluid were measured at 20 °C using a vibrating sample magnetometer (VSM) from Lake Shore, USA. The results indicated that PAO−3.5 based magnetic fluid demonstrates superparamagnetic properties under a magnetic field, showing no remanence and coercive forces. When the magnetic field strength exceeds 200 kA/m, PAO−3.5 based magnetic fluid reaches its saturation magnetization, approximately 46.7 kA/m, as depicted in Fig. 1(d).

Subsequent stability characterization involved placing PAO−3.5 based magnetic fluid on a magnet, and it remained without sedimentation for over two months. This substantiates the successful preparation of PAO−3.5 based magnetic fluid. The permanent magnets in magnetic fluid seal is N52 neodymium iron boron magnet (*φ*3 × 5 mm). The magnetic properties of the N52 magnets used in the study were also tested, revealing a residual magnetism of 1.42–1.48 T, a coercivity of at least 835 kA/m, and a maximum magnetic energy product of 390–422 kJ/m^3^.

### Rheological characterization of magnetic fluid seal

In the rheological characterization of magnetic fluid seal, several different rheological measurement fixtures are used, including coaxial cylinders, cone-and-plate, and parallel-plate fixtures. These fixtures allow for the investigation of the viscosity and yield stress of complex fluids. The coaxial cylinder fixture is suitable for low-viscosity samples and can operate under high shear rates, but its flow field is influenced by a non-linear velocity gradient distribution, which may cause significant errors when measuring non-Newtonian fluids. In contrast, the cone-and-plate fixture theoretically provides a constant shear rate, making it ideal for studying the rheological properties of non-Newtonian fluids. However, in complex fluids containing hard particles, the cone tip is prone to wear, leading to the frequent use of the parallel-plate fixture as a substitute. In the parallel-plate fixture, shear rates vary along the plate’s radial direction, necessitating corrections to the shear stress when measuring non-Newtonian fluids. The appropriate settings must be selected in the rheometer to account for this variation.

Yield stress in magnetic fluid seal arises from internal chain-like structures, the scale of which depends on the gap between the measuring fixture and the testing platform. To accurately measure the yield stress of magnetic fluid seal in a sealing gap of 0.1 mm, a parallel-plate fixture is used in this study, ensuring uniform isotropic structures within the measurement area. The rheometer characterizes the relationship between shear stress and shear rate, as well as shear stress and shear strain, to investigate the viscosity and yield stress of complex fluids^19^. Rotational rheometers, known for their precision and diverse testing modes, are widely used for rheological measurements. Their basic principle involves generating a simple shear flow field in the sample through the rotational motion of a motor and measuring the sample’s mechanical response. The fundamental physical quantities controlled and measured are the sample’s torque *M* and angular displacement$\varphi$ (or angular velocity$\Omega$), which are converted into tangential shear stress $\tau$ and shear strain $\gamma$ (or shear rate $\dot {\gamma }$) using the following relationships:

1 $$\begin{gathered} \tau ={K_\tau } \times M \hfill \\ \gamma ={K_\gamma } \times \phi \hfill \\ \end{gathered}$$

Where ${K_\tau }$ is the stress factor, and ${K_\gamma }$ is the strain factor. Both factors are related to the geometry and dimensions of the measuring fixture. Where the stress and strain factors of the plate fixture are respectively ${K_\tau }=\frac{{\text{2}}}{{\pi {R^3}}}$ and ${K_\gamma }=\frac{R}{h}$.

**Fig. 2:**
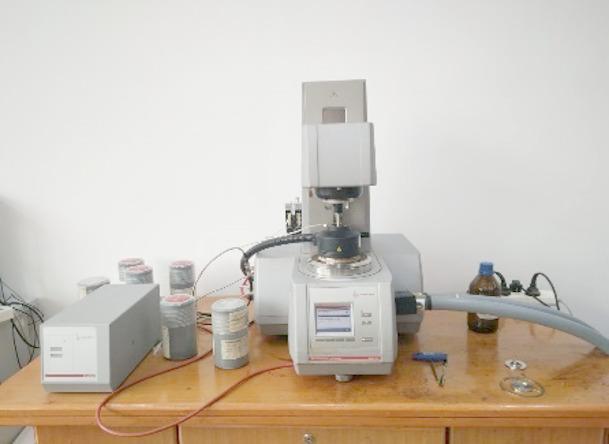
Anton Paar MCR302 rheocompass.

**Fig. 3:**
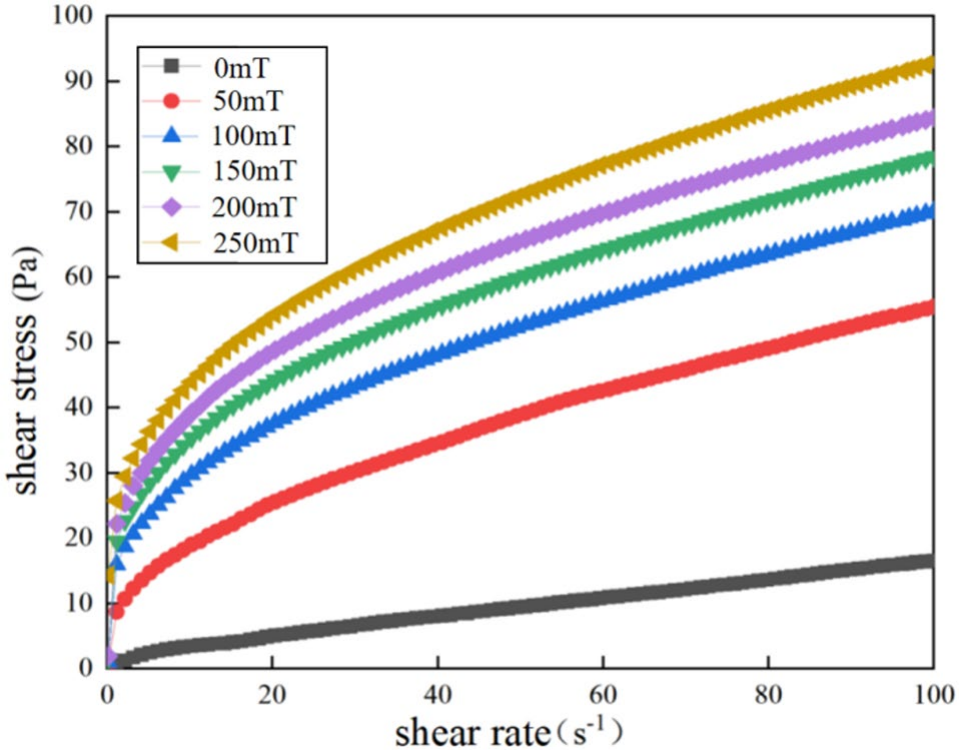
Flow curves of PAO-3.5 based magnetic fluid.

For the preparation of low-temperature magnetic fluid seals, the low-temperature-resistant magnetic fluid was formulated in Sect. "The preparations of low temperature resistant magnetic fluid". Then its rheological properties were characterized using an Anton Paar rheometer (Physica MCR 302), as shown in Fig. 2. The flow curve, stress-strain curve, and viscosity curve of the magnetic fluid were obtained. The measurement steps for the flow curve involved setting an external magnetic field, pre-shearing the fluid, measuring shear stress at different shear rates, and repeating the process while varying the magnetic field between 0 and 1.25 T. The flow curve of the low-temperature magnetic fluid is shown in Fig. 3 where the relationship between shear stress and shear rate under different magnetic field intensities is presented. It can be observed that, in the absence of a magnetic field, the shear stress and shear rate form a smooth curve, exhibiting typical Newtonian fluid behavior. Under the same conditions, the shear stress increases with the shear rate, indicating successful fluid preparation, with the magnetic particles well-coated by surfactants and evenly dispersed in the carrier liquid, showing no significant aggregation. It is worth noting that the measurement geometry used in the rheometer is a parallel-plate rotor with a diameter of 20 mm. The shear rate shown in Fig. 3 corresponds to the rotational speed of the plate rotor. Moreover, the shear rate of the magnetic fluid under rotor is directly proportional to the rotational speed of the rotor.

When a magnetic field is applied, the shear stress gradually increases, and as the magnetic field strength increases, the rate of shear stress increase stabilizes after the magnetic fluid reaches saturation magnetization. Additionally, the magnetic fluid under the external magnetic field behaves as a shear-thinning non-Newtonian fluid, with the slope of the flow curve gradually decreasing and approaching stability.

Common methods for characterizing the yield stress of magnetic fluid seal include the Herschel-Bulkley (H-B) model fitting method and the stress-strain method. The H-B model is typically used to describe shear-thinning or shear-thickening fluids with yield stress. The equation for this model is:

2 $$\tau ={\tau _{H{\text{-}}B}}(H)+k{\dot {\gamma }^n}$$

where ${\tau _{H{\text{-}}B}}(H)$ is the yield stress under magnetic field intensity *H*, *k* is the consistency coefficient, which is proportional to the fluid’s viscosity, and *n* is the flow index or power-law index. The deviation of n from 1 indicates the degree of non-Newtonian behavior of the fluid. When *n =* 1, the fluid behaves as a Bingham fluid; when *n* < 1, the fluid is shear-thinning; and when *n* > 1, the fluid is shear-thickening.

**Fig. 4:**
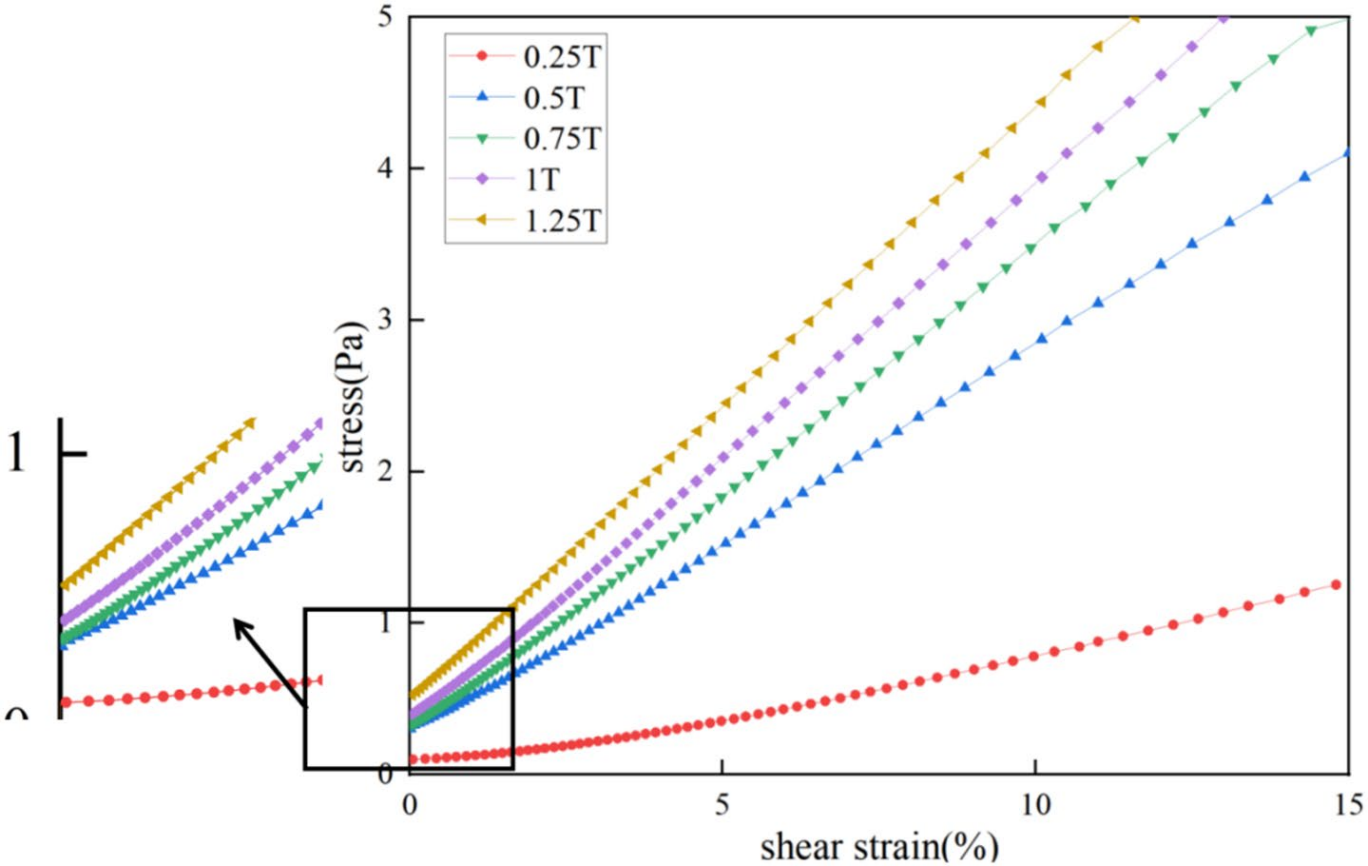
Stress strain curves of PAO-3.5 based magnetic fluid.

Since the Anton Paar MCR302 rheometer was used to characterize the yield stress of magnetic fluid seal at rest, and this rheometer is primarily a stress-controlled instrument, it differs in principle from the H-B model, which controls shear rate as the variable to obtain the corresponding curve of shear stress versus shear rate. To perform the above measurement, the rheometer must apply a large torque to the stationary rotor to overcome the rotor’s inertia and the fluid’s yield stress, allowing the rotor to move. When the applied torque is smaller than the fluid’s yield stress, the rotor rotates slightly, partially disrupting the fluid’s internal microstructure, which leads to an underestimation of the yield stress. Conversely, if the applied torque exceeds the fluid’s yield stress, the rotor accelerates, causing the yield phenomenon to be overlooked, resulting in a significant difference between the fitted yield stress value and the true value.Thus, the H-B model is more advantageous for describing the rheological behavior of fluids after they have started flowing, while the stress-strain method provides a more accurate characterization of the rheological properties of fluids with time-dependent internal structures. The stress-strain curve of low temperature resistant magnetic fluid obtained by the rheometer is shown in Fig. 4.

According to Newton’s law of viscosity, the relationship between shear stress and shear strain is given by:

3 $$\tau =\gamma \eta /t$$

Magnetic particles attract each other under the influence of dipole-dipole interactions, forming chain-like structures. Since intermolecular forces decrease as temperature increases, the strength of these chains is much higher in low-temperature environments than in high-temperature ones. Therefore, for magnetic fluid seal at rest in low-temperature conditions, the relationship between shear stress and shear strain is described by:

4 $$\tau =\gamma \eta /t+{\tau _0}$$

where ${\tau _0}$ is the yield stress of the magnetic fluid under specific conditions (Pa). From Eq. (4), it is evident that by gradually increasing the shear stress, the corresponding shear strain can be measured. Initially, if the applied shear stress is too small, it cannot overcome the yield stress of the magnetic fluid, resulting in zero or negative shear strain. As the shear stress exceeds the yield stress, the shear strain becomes positive, and the rotor starts to rotate, allowing the stress-strain curve to be recorded. The intersection of the stress-strain curve with the y-axis represents the yield stress of the magnetic fluid under these conditions. This method of measuring yield stress is called the stress-strain method.

The corresponding low-speed method, which controls shear stress, is more suited to the Anton Paar MCR302 rheometer. Here, the stress-strain method was used to characterize the yield stress of the magnetic fluid. The steps are as follows: First, the stress-strain curve of the magnetic fluid was measured without resting conditions. The measurement process involved setting the external magnetic field, applying pre-shear, measuring the shear strain under different shear stresses, and repeating the process at varying magnetic fields, ranging from 0.25 to 1.25 T, with shear stress from 0.1 to 5 Pa. The stress-strain curve for the low-temperature-resistant magnetic fluid at 20 °C after resting for 10 s is shown in Fig. 4. The curve appears as a smooth straight line, with the slope increasing with the magnetic field strength. The curve does not start from the origin, indicating the presence of a microstructure within the magnetic fluid influenced by the external magnetic field. This microstructure consists of dipole chains that resist breaking under small shear stress and strain, demonstrating that the magnetic fluid behaves as a non-Newtonian fluid with viscoelasticity in an external magnetic field, providing yield stress. The intercept on the y-axis can be considered the yield stress of the magnetic fluid under specific conditions. At low magnetic field strengths, this value increases rapidly with the magnetic field and slows down as it reaches saturation under high magnetic field strengths.

**Fig. 5:**
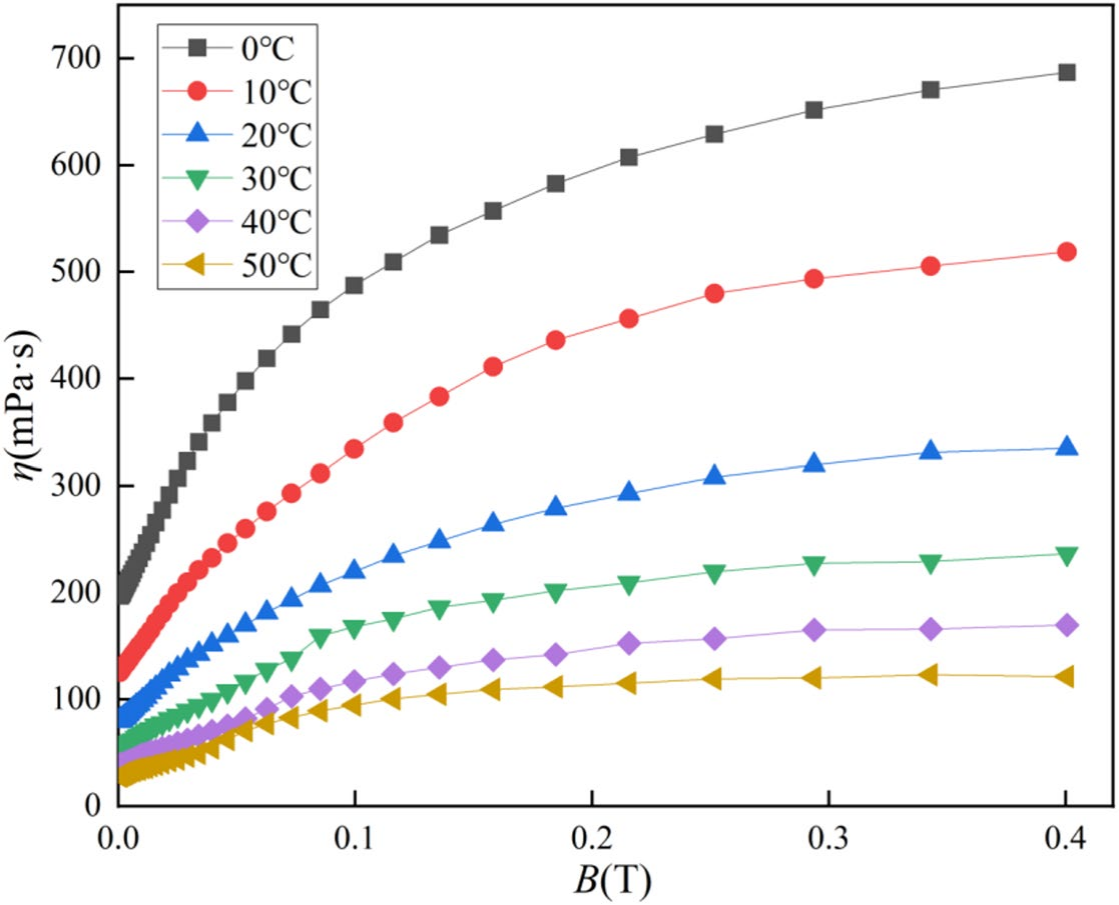
Viscosity curves with temperature and magnetic field of PAO-3.5 magnetic fluid at shear rates of 30s^− 1^.

The viscosity of the low-temperature-resistant magnetic fluid was measured at different temperatures, magnetic fields, and shear rates. Due to the limitations of the rheometer’s temperature control, the temperature range was set from 0 to 50 °C. The viscosity measurement steps were as follows: set the rheometer temperature, apply pre-shear, measure the viscosity at a specific shear rate as the magnetic field gradually increased, then change the temperature and shear rate and repeat the measurement. The magnetic field varied from 0 to 0.4 T. As shown in Fig. 5, the viscosity curve at a shear rate of 30 s⁻¹ initially increases with the magnetic field strength and then saturates at a stable value, similar to the magnetization curve of the magnetic fluid. This confirms that the magnetic particles within the fluid form a complex microstructure under an external magnetic field. Furthermore, the viscosity and its gradient increase as the temperature decreases, consistent with expectations.

## Starting torque of magnetic fluid seal

Reducing the starting torque is a major challenge for magnetic fluid seals in low-temperature environments. Due to the limitations of rheometers in characterizing the rheological properties of magnetic fluid seal—such as viscosity and yield stress—at low temperatures, it is difficult to measure these properties directly. Therefore, a general empirical formula is needed to estimate the viscosity and yield stress at specific temperatures. This allows for the prediction of whether the starting torque of a magnetic fluid seal under certain conditions meets the design requirements.

### Formula for starting torque of magnetic fluid seals

**Fig. 6:**
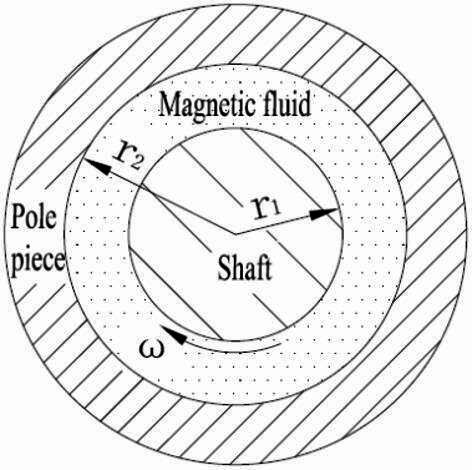
Radial section of magnetic fluid seal.

The principle of magnetic fluid seal relies on the magnetic field gradient in the seal gap, which retains the magnetic fluid in the gap, forming an “O” ring that resists external pressure and provides sealing capability. However, when the rotating shaft of the magnetic fluid seal begins to turn, the magnetic fluid in the seal gap generates frictional resistance against the shaft. As discussed in Sect. 2 on the characterization of the rheological properties of PAO-3.5 based magnetic fluid seal, yield stress develops in the magnetic fluid under a strong magnetic field, hindering the rotation of the shaft. When the shaft starts to rotate, the velocity of the magnetic fluid varies across different layers, resulting in relative motion between these layers. As shown in the radial cross-section of the magnetic fluid seal in Fig. 6, this motion creates a velocity gradient *v*, leading to internal friction between adjacent layers of fluid, which in turn generates viscous resistance.

Viscous resistance is the internal friction generated by a viscous fluid during laminar flow, opposing the flow direction. This resistance is inversely proportional to the flow gap and directly proportional to the fluid viscosity, contact area, and relative velocity. For the magnetic fluid seal gap model, where the flow gap width is *r*_2_-*r*_1_ and the gap is narrow. The radius *r*_1_ can be approximated as the flow radius and $\eta$ is the magnetic fluid viscosity. The contact area is given by *N*2π*r*_2_*l*_t_.

Where, *N* is the number of pole teeth, *l*_t_ is the width of the pole teeth (mm).

The expression for the viscous resistance at the relative motion speed *ωr*_1_ between the shaft and the pole shoe is given by:

5 $${F_\tau }=\frac{{N2\pi {\text{r}}_{1}^{2}{l_t}\eta \omega }}{{{{\text{r}}_2}-{{\text{r}}_1}}}$$

The friction torque generated by the laminar flow of the magnetic fluid is expressed as:

6 $${T_\tau }=\frac{{N2\pi {\text{r}}_{1}^{3}{l_t}\eta \omega }}{{{{\text{r}}_2}-{{\text{r}}_1}}}$$

For a stationary magnetic fluid seal, a strong magnetic field exists in the seal gap, causing the magnetic fluid to form numerous dipole chains aligned with the magnetic field. Before rotation begins, these dipole chains must be disrupted, requiring overcoming the yield stress provided by the dipole chains within the magnetic fluid. The torque due to yield stress is proportional to the contact area with the magnetic fluid, and in conjunction with Eq. (5), this torque can be expressed as:

7 $${T_{\text{s}}}=N2\pi {\text{r}}_{1}^{2}{l_t}\eta {\sigma _s}$$

Thus, the expression for the startup torque of the magnetic fluid seal is:

8 $${T_\tau }=\frac{{N2\pi {\text{r}}_{1}^{3}{l_t}\eta \omega }}{{{{\text{r}}_2}-{{\text{r}}_1}}}+N2\pi {\text{r}}_{1}^{2}{l_t}{\sigma _s}$$

### Viscosity of magnetic fluid seal

The viscosity of magnetic fluid seal with a fixed volume fraction is influenced by several factors, with three being the most significant: the viscous torque generated by the fluid’s vortex motion, changes in viscosity due to temperature, and the magnetic torque transferred to the fluid by the magnetic particles under a magnetic field. Therefore, the generalized viscosity equation for magnetic fluid seal under a uniform magnetic field can be expressed as^20^:

9 $$\frac{{\Delta \eta }}{{{\eta _{\text{c}}}}} \approx C{(\frac{{{\mu _0}mH}}{{{k_0}T}})^a}{(\frac{{{\eta _c}D}}{{{\mu _0}MH}})^b}$$

Where ${\eta _c}$ represents the viscosity of the carrier fluid at room temperature, *C* is constant number, ${k_0}$ is the Boltzmann constant, *m* represents the maximum magnetic moment of the magnetic particle. For each particle, there is $m={M_d}{V_p}$, where ${V_p}$ is the volume of each magnetic particle and ${M_d}$ is the saturation magnetization of the solid magnetic particle. This equation clearly shows the relationship between key variables affecting the viscosity of magnetic fluid seal, laying the foundation for future research on viscosity modulation and methods to reduce it.

In Sect. 2, the rheological properties of the magnetic fluid were characterized using an Anton Paar rheometer (Physica MCR 302) with a flat-plate rotor, measuring the viscosity and yield stress under different temperatures, magnetic fields, and shear rates. The gap between the rotor and the rheometer was set to 0.1 mm. Data fitting was performed using Eq. (9) and the measured data. Due to the limitations of the rheometer’s range, the temperature range was restricted to 0–50 °C.

Given the significant impact of magnetic fields, temperature, and shear rate on the viscosity of magnetic fluid seal, it is challenging to establish an initial viscosity under specific conditions. Here, we assume that the mass fraction of the magnetic fluid remains constant over a short period, meaning the initial viscosity can be treated as a constant. We replaced the viscosity ratio with a logarithmic model for fitting. Based on Eq. (9), we took the logarithm of the independent variable data and the measured magnetic fluid viscosity. A multiple regression analysis using statistical analysis software SPSS was performed, establishing an empirical formula for the viscosity of PAO-3.5 based magnetic fluid seal:

10 $${\eta _H}={\text{1}}{0^{{\text{18}}.{\text{43}}}}{B^{0.{\text{3}}0{\text{4}}}}{D^{ - 0.{\text{562}}}}{T^{ - {\text{6}}.{\text{195}}}}$$

Where *η*_*H*_ is the viscosity of the low-temperature magnetic fluid under specific conditions (mPa·s). To ensure the significance of the fitted equation, we define the range of the independent variables: magnetic field *B*, shear rate *D*, and temperature *T* are all positive values greater than zero. The goodness of fit of the multiple regression analysis R^2^ was 0.945, close to 1, with an F-statistic of 6292.407, far exceeding the 95% confidence level, and a P-value approaching 0, well below 0.05. These results indicate a high level of confidence in the data and a significant difference, demonstrating the high accuracy and good fit of the model.

### Yield stress of magnetic fluid seal

The yield stress of magnetic fluid originates from the chain-like structure formed by magnetic particles within the fluid, as explained in references^21^ and^22^. Over time, these magnetic chains experience head-to-tail aggregation and lateral aggregation, resulting in a zipper-like structure. This structure significantly thickens the chain-like formations, leading to an increase in the yield stress of the magnetic fluid. However, increasing the shear rate can disrupt these magnetic particle chains, thereby reducing the viscosity of the fluid, a phenomenon known as shear thinning. These studies suggest that for high-concentration magnetic fluid seal, the formation of magnetic chains—and thus the yield stress—can be influenced by external magnetic field strength and resting time. The ultimate yield stress *σ*_s_ of magnetic fluid can be expressed as^23^:

11 $${\sigma _\text{s}}={n_i}\frac{{3{\mu _0}}}{{2\uppi {r^4}}}{m_\text{p}}^{2}$$

Typically, for successfully prepared magnetic fluid seal, it is assumed that all magnetic particles can form chain-like structures that contribute to the yield stress. Taking Fe₃O₄-based magnetic fluid seal as an example, the magnetic moment of each particle is calculated as *M*_p_=4.5 × 10^5^ A/m. And the average diameter of the particles is 2*r*_p_ = 10^− 8^ m. The average magnetic moment of each particle is *m*_p_=-2.356 × 10^–19^ A·m^2^, where the distance between the centers of magnetic dipole particles is *r* = 2*r*_p_ = 10^− 8^ m. The number of dipole chains per unit area, *n*_*i*_, can be determined through yield stress characterization. As the external magnetic field perpendicular to d*z*d*x* increases, the number of dipole chains per unit area *n*_*i*_ also increases. For magnetic fluid seals with pressure resistance, the magnetic field in the sealing gap usually reaches the saturation magnetization required by the magnetic particles, meaning the yield stress within the gap cannot be ignored^24^. However, under certain special conditions, predicting *n*_*i*_ becomes difficult.

To obtain a general formula, Eq. (11) can be revised, with *n*_*i*_ positively correlated with temperature and resting time within a specific range. After consolidating other known variables into constants, a theoretical formula for the yield stress of magnetic fluid is derived:

12 $${\sigma _s}=C{B^x}{t^y}{T^z}$$

Similarly, a rheometer with a parallel plate rotor was used to measure the yield stress of magnetic fluid. The gap between the rotor and the rheometer was set to 0.1 mm, with variables including magnetic field, temperature, and resting time. After resting, the magnetic fluid gradually experienced increasing shear stress. At 20 °C and a resting time of 10 s, the stress-strain curve of the magnetic fluid is shown in Fig. 4. The shear stress at the smallest strain in each curve represents the yield stress under the corresponding conditions. In the absence of a magnetic field, the magnetic fluid behaves as a Newtonian fluid, so measuring yield stress without a magnetic field is irrelevant. Therefore, the magnetic field was set between 0.2 and 1.5 T, the temperature between 10 and 50 °C, and the resting time within 60 s. Statistical analysis software SPSS was used to construct an empirical formula for yield stress based on the obtained data:

13 $${\sigma _s}={\text{1}}{0^{{\text{27}}.{\text{554}}}}{B^{{\text{1}}.{\text{874}}}}{t^{0.{\text{482}}}}{T^{ - {\text{11}}.0{\text{94}}}}$$

Here, *B* represents the magnetic field, *T* represents the temperature, and *t* represents the resting time. All variables are positive numbers. The goodness-of-fit R^2^ of the multiple regression analysis was 0.897; the F-statistic was 358.058, far exceeding the 95% confidence level; and the P-value was approximately zero, well below 0.05, indicating high confidence in the fitting results, significant differences in the data, and high precision in the analysis. From the two empirical formulas concerning the rheological properties of magnetic fluid seal, we can deduce the effects of magnetic field and temperature on the viscosity and yield stress of magnetic fluid seal. These formulas also suggest that adjusting the magnetic field can effectively influence the viscosity and shear stress of magnetic fluid seal, supporting the practical value of controlling the magnetic field in magnetic fluid seals to manage starting torque.

The fitting results for viscosity and yield stress reveal that the magnetic field, shear rate, and temperature all affect the viscosity and yield stress of magnetic fluid seal. The standardized coefficients of temperature’s impact on viscosity and yield stress are 0.381 and 0.258, respectively, indicating a significant effect of temperature on viscosity. The corresponding standardized coefficients for the magnetic field are 0.758 and 0.803, suggesting a greater influence of the magnetic field than temperature. This shows that adjusting the magnetic field can significantly affect the viscosity and yield stress of magnetic fluid seal. It also implies that adjusting the magnetic field in the sealing gap can modulate the friction and starting torque of the magnetic fluid seal.

Using Eqs. (10) and (11), the viscosity and yield stress of magnetic fluid under different conditions can be estimated. For instance, at -55 °C, 0.5 T and 3 h resting time, the viscosity of PAO-3.5 based magnetic fluid is estimated to be 27.404 Pa·s, and the yield stress is 1441.388 Pa. By substituting known data into the torque formula (8), the starting torque of the low-temperature-resistant magnetic fluid seal at -55 °C after 3 h of resting was calculated to be 0.1128 N·m at a rotational speed of 0.1 r/s.

## Low-temperature starting torque test of magnetic fluid seal

### The procedures and results of starting torque test

**Fig. 7:**
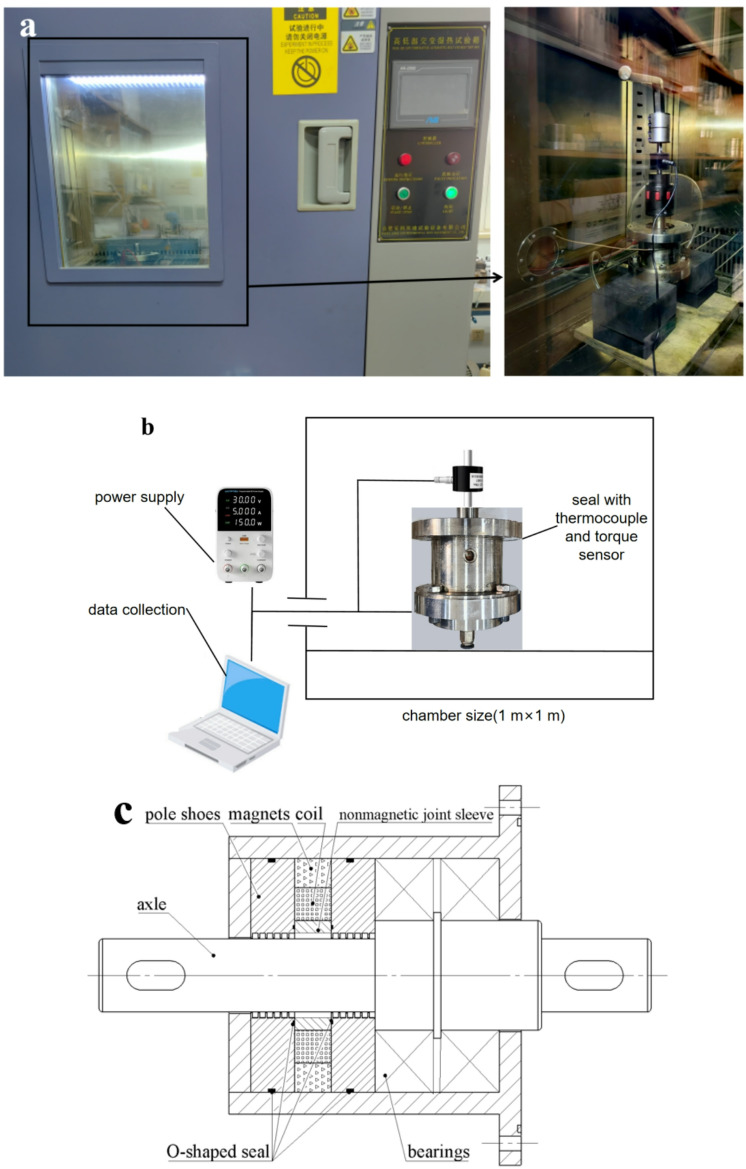
**a)** High and low temperature experimental box, **b**) Internal schematic diagram of High and low temperature experimental box, **c**) Seal structure.

**Fig. 8:**
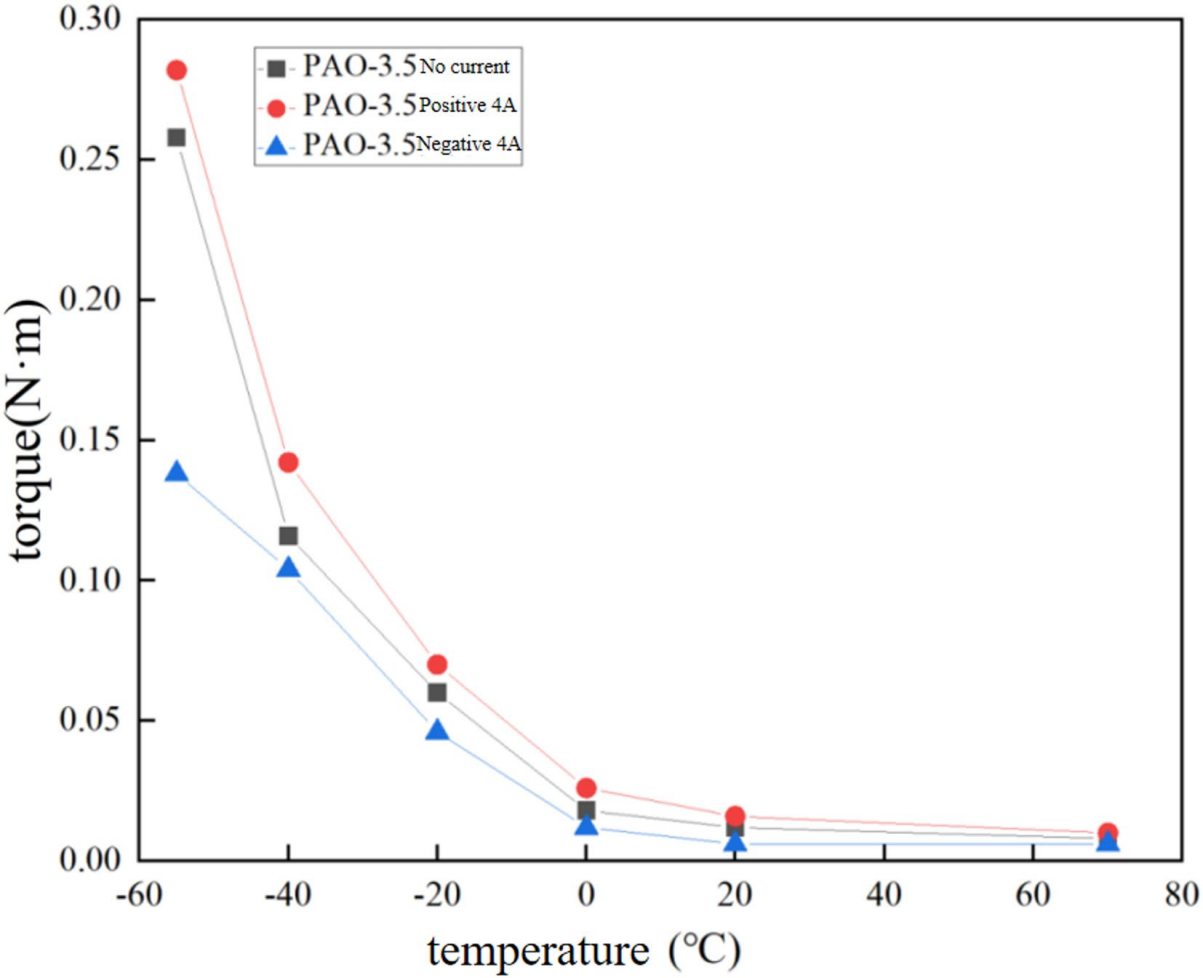
Starting torque experiment in low temperature range.

The assembled sealing device is placed in a temperature chamber for cooling. The bearings of the sealing device in the low-temperature environment is ceramic bearings. The temperature chamber is connected to the air compressor, thermocouple, DC power supply, pressure sensor, and torque sensor through a communication channel with the external environment. Cork plugs are used to isolate heat conduction between the inside and outside of the temperature chamber, as shown in Fig. 7(a). Blind holes on the pole shoes allow the needle thermocouple to measure the temperature near the pole teeth, providing a more accurate measurement of the actual temperature of the magnetic fluid in the sealing gap. The torque value is obtained from a static torque sensor. The schematic diagram of chamber and experimental setup for measuring torque is shown in Fig. 7(b). To evaluate the influence of magnetic field on the starting torque of magnetic fluid seal, the magnet type in seal is a combined magnetic source of electromagnet and permanent magnet. The seal structure is shown in Fig. 7(c). The torque sensor is ZNNT-J3 torque sensor made by Chino Sensor, whose measuring range is 0 ~ 1 N·m and accuracy is less than 0.02%. The shaft of seal is 25 mm, and the seal gap is 0.1 mm. The torque of the sealing device without magnetic fluid at room temperature is 0.004 N·m, and the torque of the sealing device without magnetic fluid after being left at -55 °C for 3 h is 0.0212 N·m. Due to the limitations of the rheometer’s measurement range, it is impractical to measure the mass fraction and viscosity of the magnetic fluid at-55 °C. Considering the focus of the experiment and to accurately describe the results, reasonable assumptions were made: (1) The mass fraction and magnetization intensity of the magnetic fluid in the sealing gap remain constant during temperature changes. (2) The pole teeth and shaft are made of uniform material with uniform thermal conductivity. (3) The magnetic fluid is fully filled into the sealing gap, and the uneven heat transfer caused by the non-uniform filling of the magnetic fluid is ignored. Subsequently, a low-temperature starting torque test was conducted on the magnetic fluid sealing filled with PAO-3.5-based magnetic fluid. The steps for measuring the starting torque at low temperatures are as follows: First, use the quantitative filling method of magnetic fluid to inject it into the magnetic fluid seal to measure and confirm that the pressure resistance value has been reached before considering the injection successful. Next, place the sealed device in a temperature chamber and set the environmental temperature. Once the temperature reaches the set point, maintain it until the temperature sensor shows a change rate of less than 0.1 °C/s. Then, let the device sit at this temperature for 3 h. After 3 h, power the electromagnet as required, open the door of the temperature chamber, and to prevent the main shaft from rotating before torque measurement, hold the main shaft to prevent it from turning. Quickly install the static torque sensor. Then, slowly rotate the main shaft clockwise for 360°, taking about 10 s to complete the measurement of the starting torque. Afterward, rotate the main shaft counterclockwise for 360° to measure the friction torque during normal operation. To facilitate reading, the static torque sensor is set to peak mode. The entire process should be completed within 1 min, with the temperature sensor showing a temperature increase of less than 2 °C. For the control group that requires power supply to the electromagnet, adjust the sealing gap magnetic field by controlling the power supply current, which is a critical variable. Since the electromagnet has a high heating power, it will cause the temperature of the magnetic fluid to rise rapidly after activation, so the power supply time is a critical variable and should be controlled before opening the low-temperature chamber door. Each test should be repeated three times to obtain an average result to ensure accuracy. Then, the low temperature domain magnetic liquid start torque experiment was carried out for the low temperature magnetic liquid. The steps of the low temperature start torque experiment were repeated, and the magnetic fluid sealing start torque was measured successively at-55, -40, -20,0, 20 and 70 °C. The experimental results are shown in Fig. 8. It is worth noting that in Fig. 8, positive or negative currents respectively represent magnetic fields generated by the electromagnet that are aligned with or opposite to the direction of the permanent magnet’s field. Based on simulations of magnetic field the sealing gap, the magnetic flux density gradients at a single-pole tooth were calculated to be 0.5 T for a positive 4 A current, 0.05 T for a negative 4 A current, and 0.3 T with no current conditions.

## Discussion

The experimental results of the starting torque for magnetic fluid seals at low temperatures are shown in Fig. 8. These results clearly demonstrate the close relationship between the starting torque and both the ambient temperature and the magnetic field strength. The starting torque decreases as the temperature rises. Specifically, when the temperature increases from − 55 °C to 0 °C, the starting torque drops rapidly. However, as the temperature continues to rise above 0 °C, the drop rate slows down or even stops, indicating that a significant portion of the starting torque comes from the frictional torque of the bearings. Since ceramic bearings are self-lubricating, their frictional torque remains relatively stable with changes in temperature. The additional torque provided by the ceramic bearings in the magnetic fluid seal torque does not vary with temperature changes. Therefore, the influence of the bearing’s friction torque can be neglected. The trend suggests that the starting torque and temperature have an approximate relationship with a negative constant power function, which validates the empirical formulas of magnetic fluid viscosity (10) and yield stress (13). It is also found that the starting torque is proportional to the saturation magnetization intensity or yield stress of the magnetic fluid. The saturation magnetization intensity is approximately a positive constant power function of the magnetic field strength in the sealing gap. This confirms the correctness of the empirical formulas (10) and (13), which state that the viscosity and yield stress are positively related to the magnetic field strength as a positive constant power function. The experimental results show that the starting torque of magnetic fluid seal at 55 °C without current is 0.262 N·m after 3 h of standing, which is close to 0.1128 N·m calculated by empirical formula and torque formula. The differences between the experimental and theoretical values can be attributed to several factors. First, the derivation of the empirical formula involves certain simplifications that may reduce its accuracy. Second, measurement errors may arise from the rheometer’s precision and methodology. Third, wear and tear on the sealing device during manufacturing, installation, and testing can also affect performance. Lastly, uneven injection of the magnetic fluid beneath the pole teeth contributes to the observed discrepancies.

## Conclusion

The rheological properties of magnetic fluid seal, including flow curves, stress-strain curves, and viscosity curves, were characterized using a rheometer. The flow curve shows a smooth curve between shear stress and shear rate under zero magnetic field, exhibiting typical Newtonian fluid behavior, indicating good dispersion of magnetic particles in the carrier liquid without agglomeration. Under an external magnetic field, the fluid exhibited shear thinning, characteristic of a non-Newtonian fluid. Meanwhile, the rheological characterization on the standing time proves that the standing time highly influences the yield stress of magnetic fluid. The stress-strain curve was a smooth straight line with an intercept on the vertical axis, demonstrating the viscoelastic properties of magnetic fluid under an external magnetic field and confirming the existence of dipole chain structures. The viscosity curve showed that the viscosity of the magnetic fluid initially increased with the magnetic field strength and eventually saturated at a stable value, similar to the magnetization curve of the fluid. These characterizations of the rheological properties confirm the formation of complex microstructures, such as dipole chain structures, within the magnetic fluid under an external magnetic field.

Based on the torque model for magnetic fluid seals, it was found that the starting torque of the seal is composed of the friction torque and yield stress of the magnetic fluid in the sealing gap. These two forces are respectively proportional to the viscosity and yield stress of the fluid. Consequently, the starting torque formula for the magnetic fluid seal was derived. By correlating the viscosity and torque expressions with external influencing factors and combining the rheological properties of the magnetic fluid, empirical formulas for the viscosity and yield stress of magnetic fluid were fitted using statistical analysis software SPSS.

The verification through low-temperature start-up torque tests shows that the start-up torque of magnetic fluid seals decreases as temperature rises and the magnetic field strength decreases, with the rate of change in start-up torque slowing down as temperature increases. The start-up torque test confirms the accuracy of the empirical formulas for magnetic liquid viscosity and yield stress. These empirical formulas can be used to estimate the viscosity, yield stress, and starting torque of magnetic fluid seals under target working conditions. Theoretical calculations provide a preliminary assessment of whether the designed PAO-3.5 based magnetic fluid seal meets the requirements, and the results can be further validated through torque experiments.

Further research is needed to refine the derivation of empirical formulas, improve the measurement accuracy of rheometers, and enhance the machining precision of sealing components. Additionally, using new parts can help reduce wear, and optimizing the method of injecting magnetic fluid will lead to better prediction accuracy of the starting torque in magnetic fluid seals.

## Data Availability

Data available on request from the author Jiawei Liu.
